# Knockdown of hsa_circ_0023028 inhibits cell proliferation, migration, and invasion in laryngeal cancer by sponging miR-194-5p

**DOI:** 10.1042/BSR20190177

**Published:** 2019-06-14

**Authors:** Xiaofeng Chen, Xiaoqing Su, Chuansai Zhu, Jing Zhou

**Affiliations:** Department of Otolaryngology, Ruian People’s Hospital, No. 108 Wansong Road, Yuhai Street, Ruian City 325200, Zhejiang Province, China

**Keywords:** competing endogenous RNA, hsa_circ_0023028, laryngeal cancer, miR-194-5p

## Abstract

Emerging evidences have proposed that circular RNAs (circRNAs) play a major role in carcinogenesis. Hsa_circ_0023028 has been reported to be aberrantly expressed in laryngeal cancer (LCa). However, the role and the mechanism of hsa_circ_0023028 in LCa have not been adequately studied. In the present study, we demonstrated that hsa_circ_0023028 expression was up-regulated in LCa tissues and cell lines. miR-194-5p was down-regulated in LCa cells. Functionally, knockdown of hsa_circ_0023028 inhibited the proliferation, migration, and invasion of LCa cells, as evidenced by the reduced number of 5-Ethynyl-2′-deoxyuridine (EdU)-positive cells and decreased number of migrated and invaded cells. Additionally, hsa_circ_0023028 was identified as an miR-194-5p sink. A negative correlation between miR-194-5p and hsa_circ_0023028 expression was observed in LCa tissues. Besides, down-regulation of miR-194-5p attenuated the inhibitory effects of hsa_circ_0023028 silencing on LCa cell proliferation, migration, and invasion. In summary, hsa_circ_0023028 functions as an miR-194-5p sponge to promote the proliferation, migration, and invasion of LCa cells.

## Introduction

Laryngeal cancer (LCa) is one of the most frequently encountered malignancy of the head and neck that affected more than 1.4 million people worldwide in 2015 [1]. Laryngeal squamous cell carcinoma (LSCC) is the most common type of LCa. LCa usually arises from the skin of the larynx. It is the eighth leading cause of cancer-related deaths worldwide, and caused over 105900 deaths in 2015 [2]. LCa clinically presents with hoarseness, breathing difficulties, persistent cough, and dysphagia. Family history, tobacco smoking, and alcohol consumption are considered to be important contributors to LCa. Stages I and II of LCa are often curable with surgery and radiotherapy, alone or in combination. While patients with advanced LCa (stages III and IV) are always treated with surgery and chemotherapy (alone or in combination), the prognosis is unsatisfactory [3]. Clinically, metastasis is regarded as the major obstacle in the treatment of patients with LCa, but the molecular mechanism of LCa progression is still unclear. Therefore, a detailed understanding of the mechanism involved in LCa progression is crucial for developing a novel and effective therapy for LCa.

Noncoding RNAs (ncRNAs) are a class of endogenous RNAs that are not translated into proteins. ncRNAs occupy more than 90% of the human genome and have become a major focus of research [4]. ncRNAs participate in the regulation of inflammatory signaling that underlie many diseases [5]. Abundant and functionally important types of ncRNAs include long ncRNAs, small ncRNAs, tRNAs, rRNAs, and circular RNAs (circRNAs). circRNAs are considered as a novel type of ncRNAs that are hallmarked by covalently closed continuous loops [6]. With the rapid development of next-generation sequencing, an increasing number of circRNAs has been discovered. circRNAs are widely represented in the eukaryotic transcriptome and are generated by back-splicing without the 3′ and 5′ ends [7,8]. Owing to their unique structure, circRNAs are able to counteract RNA exonucleolytic digestion, which makes them an ideal biomarker for disease diagnosis [9]. Recently, increasing evidence supports the notion that dysregulated circRNAs are tightly linked to the progression of human diseases [10]. circRNAs have been shown to function as key regulators of carcinogenesis. The expression of circRNAs in cancers has been found to be closely correlated with clinical features, such as tumor-node-metastasis stage, metastasis, and tumor size [11]. Furthermore, circRNAs have been reported to be implicated in the progression of human cancers by modulating the proliferation, invasion, apoptosis, and cell cycle of tumor cells [12]. Hsa_circ_0023028, a novel identified circRNA, has been reported to be located at chr11:66515849-66590145 in gene symbol C11orf80 and markedly overexpressed in LSCC tissues. Moreover, hsa_circ_0023028 level in LSCC tissues was shown to be closely related with the clinical characteristics of patients with LSCC, such as tumor grade, lymph node metastasis, primary location, and clinical stage [13]. However, very little information is available regarding the role of hsa_circ_0023028 in the occurrence and development of LCa.

microRNAs (miRNAs) are a type of small (∼21 nucleotides) ncRNAs that inhibit the translation of messengers RNAs (mRNAs), and have been implicated in a cohort of biological processes [14]. After nuclear processing, miRNA precursors are exported from the nucleus and form mature miRNAs in the cytoplasm. miRNAs assemble into the RNA-induced silencing complex and base-pair with the 3′-untranslated region (UTR) of their target mRNAs, leading to mRNA degradation and/or impairment of translation [15]. Alternatively, miRNAs can bind competitively to the AU-rich regions in the target mRNA 3′-UTR and inhibit the degradation of target mRNAs, thereby mediating mRNA up-regulation [16]. Emerging evidence suggests that miRNAs serve as key players in carcinogenesis [17]. Aberrant expression of miRNAs was reported in numerous malignancies, including LCa [18]. miR-194-5p, a vertebrate-specific miRNA, plays an important role in the progression of human cancers, including gastric cancer [19], hepatocellular carcinoma [20], LSCC [21], and gallbladder cancer [22]. Up-regulation of miR-194-5p was shown to cause a marked reduction in cell proliferation, suggesting its anti-tumor effect in gallbladder cancer [22]. However, little attention has been given to the biological function and underlying mechanism of miR-194-5p in LCa tumorigenesis.

Here, we aimed to explore the biological role of hsa_circ_0023028 in LCa, and identified hsa_circ_0023028 as an miR-194-5p sponge in carcinogenesis. Moreover, hsa_circ_0023028 promoted the proliferation and metastasis of LCa cells by sponging miR-194-5p. These findings suggest that hsa_circ_0023028 may possibly serve as a promising therapeutic target for LCa.

## Materials and methods

### Patient samples

Twenty pairs of liquid nitrogen frozen LCa solid tumors and their corresponding adjacent noncancerous tissues were obtained from the Department of Otolaryngology, Ruian People’s Hospital. Patients who participated in the present study were confirmed by pathological examination and did not receive any adjuvant therapy prior to operation. Protocols were approved by the Ethics Committee of Ruian People’s Hospital and informed consent was obtained from all patients prior to participation in the present study.

### Cell culture

Human LCa cells (Hep-2 and TU212) and normal nasopharyngeal epithelial cell line (NP69) were obtained from the Cell Resource Center of Shanghai Institutes for Biological Sciences, Chinese Academy of Sciences (Shanghai, China). Cells were maintained in RPMI-1640 medium supplemented with 10% fetal bovine serum (FBS; Invitrogen, Grand Island, NY, U.S.A.) and 1% penicillin/streptomycin (Solarbio, Beijing, China) in a humidified atmosphere of 95% air and 5% CO_2_ at 37°C.

### Cell transfection

Small interfering RNA (siRNA) targeting hsa_circ_0023028 (si-circ), miR-194-5p mimic, miR-194-5p inhibitor, and matched controls were synthesized by GenePharma (Shanghai, China). Cell transfection was carried out using Lipofectamine 2000 reagent (Invitrogen) as per the manufacturer’s instructions.

### Detection of cell proliferation capacity

Cell proliferation was evaluated by Cell Counting Kit-8 (CCK-8) and 5-Ethynyl-2′-deoxyuridine (EdU) assays. For CCK-8 assay, Hep-2 and TU212 cells were seeded into 96-well plates and incubated overnight in a humidified atmosphere of 95% air and 5% CO_2_ at 37°C. At the indicated time points of 0, 24, 48, and 72 h post-transfection, the transfected cells were incubated in RPMI-1640 medium containing CCK-8 reagent (10 μl; Solarbio). After 2 h of incubation in a 5% CO_2_ incubator at 37°C, the absorbance at 450 nm was measured using a NanoDrop spectrophotometer (Thermo Fisher Scientific, Wilmington, DE, U.S.A.).

For EdU assays, Hep-2 and TU212 cells were transfected with siRNA specific for hsa_circ_0023028 (si-circ), miR-194-5p inhibitor, or siRNA negative control (si-NC) and incubated for 48 h in 24-well plates. Following this, cells were incubated with 10 µM EdU (Beyotime, Shanghai, China) for 2 h, followed by fixation in 4% paraformaldehyde for 15 min. After washing, cells were permeated with Triton X-100 for 10 min, and then incubated with the reaction mixture for 30 min. Thereafter, cells were stained with Hoechst 33342 for 5 min in darkness. The number of EdU-positive cells was counted under a fluorescence microscope.

### Transwell migration and invasion assay

Transwell chambers (Corning, Steuben County, NY, U.S.A.) were used to assess the migration and invasion of Hep-2 and TU212 cells. Hep-2 and TU212 cells were transfected with si-circ, miR-194-5p inhibitor, or si-NC. At 48 h post-transfection, Hep-2 and TU212 cells were harvested and then seeded in the upper chambers pre-coated with fibronectin (for migration assay; Solarbio) or Matrigel (for invasion assay; BD Biosciences, Franklin Lakes, NJ, U.S.A.). The lower chambers were filled with RPMI-1640 medium containing 5% FBS. After 48 h of incubation at 37°C, the migrated and invaded cells were fixed in 4% paraformaldehyde and stained with 0.1% Crystal Violet (Solarbio) for 4 min. The number of cells that migrated or invaded to the lower side of the membrane was counted in five random fields using a light microscope.

### Luciferase reporter assay

The hsa_circ_0023028 sequence containing wild-type or mutated miR-194-5p binding site (WT-circ; MUT-circ) was fused into pmirGLO vector (Promega, Madison, WI, U.S.A.). The luciferase reporter vector was co-transfected into Hep-2 and TU212 cells with miR-194-5p or miRNA negative control (miR-NC). The Dual-Luciferase Reporter Assay System (Promega) was used to detect the luciferase activities at 48 h post-transfection as per the manufacturer’s specifications.

### Quantitative real-time polymerase chain reaction

RNA extraction from the tissues and cells was performed using TRIzol reagent (Invitrogen) and the RNA concentration was assessed using a spectrophotometer. For miRNAs, TaqMan miRNA assay (Applied Biosystems, Foster City, CA, U.S.A.) was applied as per the manufacturer’s instructions. miRNA and U6 primers were purchased from Applied Biosystems. For hsa_circ_0023028, SuperScript III Reverse Transcriptase (Life Technologies, Gaithersburg, MD, U.S.A.) and Power SYBR Green Master Mix (Life Technologies) were used for cDNA synthesis and quantitative real-time polymerase chain reaction (qRT-PCR), respectively. qRT-PCR was performed using the CFX96 Real-time PCR system (Bio-Rad, Hercules, CA, U.S.A.). The primers’ sequences used were as follows: hsa_circ_0023028: forward, 5′-GCC AAG GCT CAG CAG AAA CTA-3′ and reverse, 5′-TGT TGC TCC AAG ACC TTG TCC-3′; glyceraldehyde-3-phosphate dehydrogenase (GAPDH): forward, 5′-GGG AAA CTG TGG CGT GAT-3′ and reverse, 5′-GAG TGG GTG TCG CTG TTG A-3′; miR-194-5p: forward, 5′-GCG GCG GTG TAA CAG CAA CTC C-3′ and reverse, 5′-ATC CAG TGC AGG GTC CGA GG-3′; miR-145: forward, 5′-GTC CAG TTT TCC CAG GAA TCC CT-3′ and reverse, 5′-GCT GTC AAC ATA CGC TAC GTA ACG-3′; miR-1225-5p: forward, 5′-GTG GGT ACG GCC CAG T-3′ and reverse, 5′-CAC AGG GGC TCA GTC AGT C-3′; miR-1234: forward, 5′-GTG AGT GTG GGG TGG CTG-3′ and reverse, 5′-GGT GGT CAG GCC GAC TAG G-3′; miR-186: forward, 5′-AAG AAT TCT CCT TTT GGG CT-3′ and reverse, 5′-GTG CGT GTC GTG GAG TCG-3′; miR-433: forward, 5′-CTG GTA GGA TCA TGA TGG GAT-3′ and reverse, 5′-TCA ACT GGT GTC GTG GAG T-3′; U6: forward, 5′-GCT TCG GCA CAT ATA CTA AAA T-3′ and reverse, 5′-CGC TTC ACG AAT TTG CGT GTC AT-3′. Expression levels were normalized to GAPDH for hsa_circ_0023028 and U6 for miRNAs. The 2^–ΔΔ*C*^_t_ method was applied to determine the expression of hsa_circ_0023028 and miRNAs.

### Statistical analysis

All experiments were performed in triplicate and statistical analysis was carried out using SPSS 20.0 software (IBM, Armonk, NY, U.S.A.). The results are expressed as mean ± standard error of the mean, and data were analyzed using Student’s *t* test or one-way analysis of variance. A *P*-value less than 0.05 was defined as the level of significance.

## Results

### Ectopic expression of hsa_circ_0023028 in LCa tissues and cell lines

**Figure 1:**
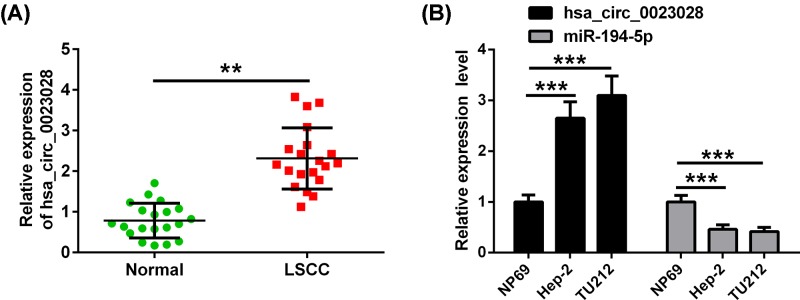
The expression of hsa_circ_0023028 characterizes LCa tissues and cell lines (**A**) A total of 20 LCa solid tumor samples and paired adjacent noncancerous tissue samples were obtained from patients with LCa. Hsa_circ_0023028 expression was detected to be up-regulated in LCa tissues using qRT-PCR. (**B**) Expression levels of hsa_circ_0023028 and miR-194-5p were validated in human LCa cell lines (Hep-2 and TU212) and normal nasopharyngeal epithelial cell line (NP69) using qRT-PCR. **P<0.01 and ***P<0.001.

Previously, microarray analysis showed that hsa_circ_0023028 was up-regulated in LSCC [12]. LCa tissues and paired adjacent noncancerous tissues were collected to evaluate the expression of hsa_circ_0023028 in LCa. qRT-PCR analysis revealed that hsa_circ_0023028 was highly expressed in LCa tissues as compared with their adjacent noncancerous tissues (Figure 1A). In line with this, the expression level of hsa_circ_0023028 was found to be higher, but, miR-194-5p was lower in Hep-2 and TU212 cells than that in NP69 cells (Figure 1B).

### Knockdown of hsa_circ_0023028 inhibits the proliferation of LCa cells

**Figure 2:**
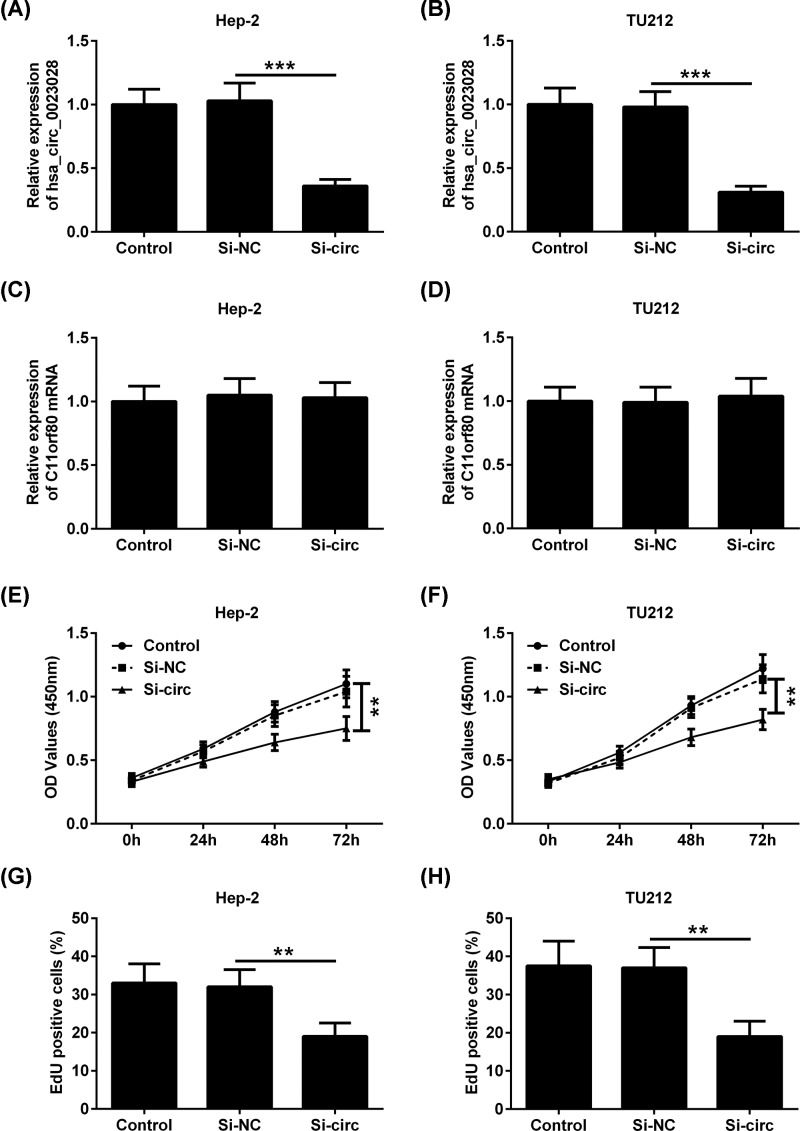
Knockdown of hsa_circ_0023028 inhibits the proliferation of LCa cells Hep-2 and TU212 cells were transfected with si-NC or si-circ. Cells were collected at 48 h after transfection. (**A,B**) qRT-PCR analysis of hsa_circ_0023028 expression in Hep-2 and TU212 cells. (**C,D**) CCK-8 assay showed the viability of Hep-2 and TU212 cells transfected with si-NC or si-circ. CCK-8 (**E,F**) and EdU (**G,H**) proliferation assay showed the cell proliferation ability of Hep-2 and TU212 cells transfected with si-NC or si-circ. **P<0.01 and ***P<0.001.

Since hsa_circ_0023028 is up-regulated in LCa, we knocked down hsa_circ_0023028 expression to assess its functional role in LCa. A specific siRNA targeting hsa_circ_0023028 was transfected into Hep-2 and TU212 cells, leading to down-regulation of hsa_circ_0023028 (Figure 2A,B). We found that down-regulation of hsa_circ_0023028 significantly reduced the viability of Hep-2 and TU212 cells compared with the control group (Figure 2C,D). In parallel, silencing of hsa_circ_0023028 in Hep-2 and TU212 cells resulted in a striking reduction in cell proliferation, as indicated by the CCK-8 (Figure 2E,F) and EdU assays (Figure 2G,H).

### Knockdown of hsa_circ_0023028 inhibits the migration and invasion of LCa cells

**Figure 3:**
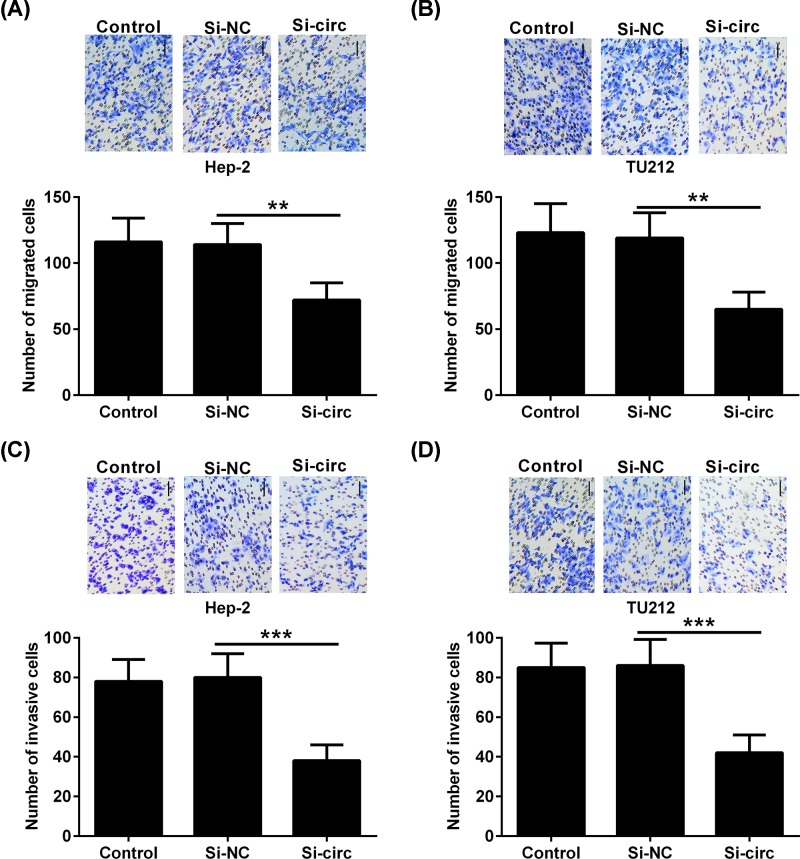
Knockdown of hsa_circ_0023028 inhibits the migration and invasion of LCa cells A specific siRNA targeting hsa_circ_0023028 was transfected into Hep-2 and TU212 cells, and cells were collected at 48 h after transfection. (**A,B**) The migration ability of Hep-2 and TU212 cells transfected with si-circ or si-NC was determined using transwell migration assay. (**C,D**) Transwell invasion assay was performed to evaluate the invasion ability of Hep-2 and TU212 cells transfected with si-circ or si-NC. **P<0.01 and ***P<0.001.

To further elucidate the biological functions of hsa_circ_0023028 in LCa, a specific siRNA targeting hsa_circ_0023028 was used to down-regulate its expression in Hep-2 and TU212 cells, followed by transwell migration and invasion assays. As shown in Figure 3A,B, knockdown of hsa_circ_0023028 inhibited the cell migration ability of Hep-2 and TU212 cells compared with the control group. Meanwhile, compared with the control group, transfection of si-circ into Hep-2 and TU212 cells caused a marked reduction in cell invasion (Figure 3C,D).

### Knockdown of hsa_circ_0023028 up-regulates the expression of miRNAs in LCa cells

**Figure 4:**
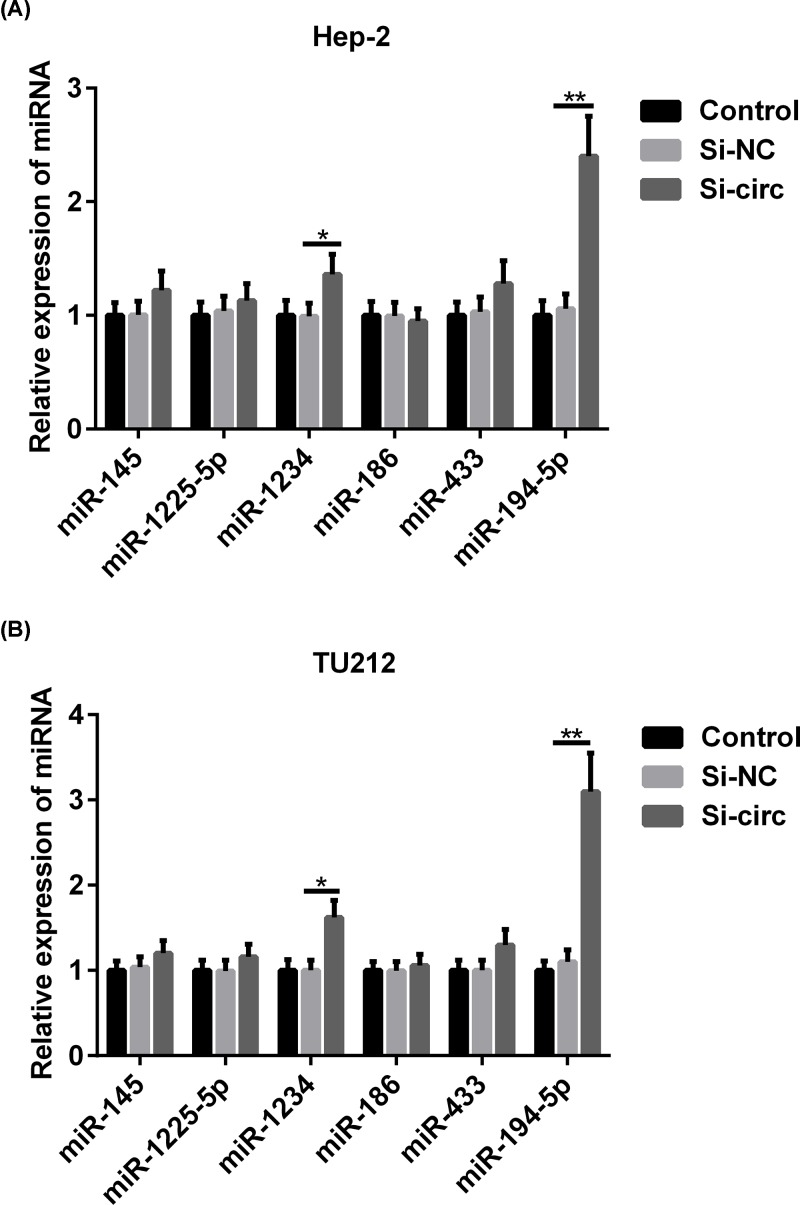
Knockdown of hsa_circ_0023028 up-regulates the expression of miR-194-5p in LCa cells A specific siRNA targeting hsa_circ_0023028 was transfected into Hep-2 and TU212 cells, and cells were harvested at 48 h post-transfection. (**A,B**) qRT-PCR analysis of miR-194-5p expression after transfecting Hep-2 and TU212 cells with si-NC or si-circ. *P<0.05 and **P<0.01.

Bioinformatics analysis that predicted the potential targets of hsa_circ_0023028 were miR-145, miR-1225-5p, miR-1234, miR-186, miR-433, and miR-194-5p, which share complementary binding sites with hsa_circ_0023028. To investigate the mechanisms that mediate the effects of hsa_circ_0023028 in LCa, we measured the expression level of miR-145, miR-1225-5p, miR-1234, miR-186, miR-433, and miR-194-5p in control and hsa_circ_0023028-silenced Hep-2 and TU212 cells. We found that the expression of miR-1234 and miR-194-5p was strikingly increased, but that of miR-145, miR-1225-5p, miR-1234, miR-186, and miR-433 did not show significant changes in hsa_circ_0023028-silenced Hep-2 cells. Of note, the expression levels of miR-194-5p and miR-1234 were obviously increased in the si-circ group, especially the expression of miR-194-5p (Figure 4A). Similar results were also seen in TU212 cells (Figure 4B). Therefore, in subsequent experiments, we focused on the role of miR-194-5p due to its marked up-regulation in hsa_circ_0023028-silenced Hep-2 and TU212 cells.

### Hsa_circ_0023028 serves as a sponge for miR-194-5p

**Figure 5:**
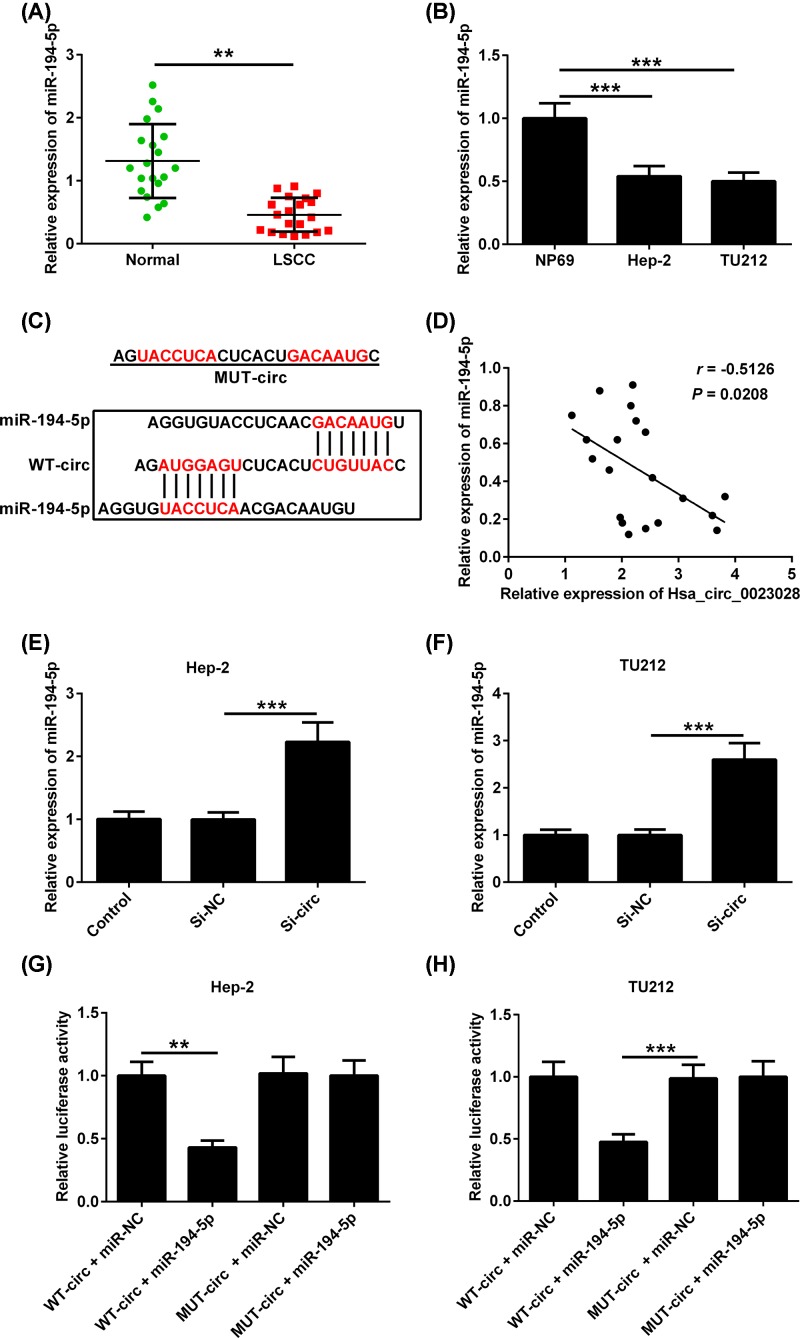
Hsa_circ_0023028 serves as a sponge for miR-194-5p (**A,B**) The expression of miR-194-5p was evaluated in LCa tissues and cell lines using qRT-PCR. (**C**) The complimentary binding site for miR-194-5p in the hsa_circ_0023028 sequence is shown. (**D**) Correlation analysis of hsa_circ_0023028 and miR-194-5p expression in LCa tissues. (**E,F**) qRT-PCR analysis of miR-194-5p level after transfecting Hep-2 and TU212 cells with si-NC or si-circ. (**G,H**) Luciferase reporter assay showed the luciferase activity of Hep-2 and TU212 cells co-transfected with WT-circ or MUT-circ and miR-194-5p or miR-NC. **P<0.01 and ***P<0.001.

The expression of miR-194-5p was lower in LCa tissues than that in their adjacent noncancerous tissues (Figure 5A). Consistently, miR-194-5p expression was down-regulated in LCa cell lines (Hep-2 and TU212) compared with that in NP69 cells (Figure 5B). As indicated in Figure 5C, the sequence of hsa_circ_0023028 harbors a putative miR-194-5p binding site. Additionally, a negative correlation between hsa_circ_0023028 and miR-194-5p expression was observed in LCa tissues (Figure 5D). Notably, down-regulation of hsa_circ_0023028 by si-circ obviously increased the expression level of miR-194-5p in Hep-2 and TU212 cells (Figure 5E,F). To further validate the association between hsa_circ_0023028 and miR-194-5p, we performed luciferase reporter assay in Hep-2 and TU212 cells. Results revealed that the luciferase activity of WT-circ was reduced in the presence of miR-194-5p, but remained unaltered in the presence of miR-NC. Simultaneously, transfection with miR-194-5p or miR-NC did not affect the luciferase activity of MUT-circ in Hep-2 and TU212 cells (Figure 5G,H).

### Down-regulation of miR-194-5p attenuates the inhibitory effect of hsa_circ_0023028 silencing on LCa cell proliferation, migration, and invasion

**Figure 6:**
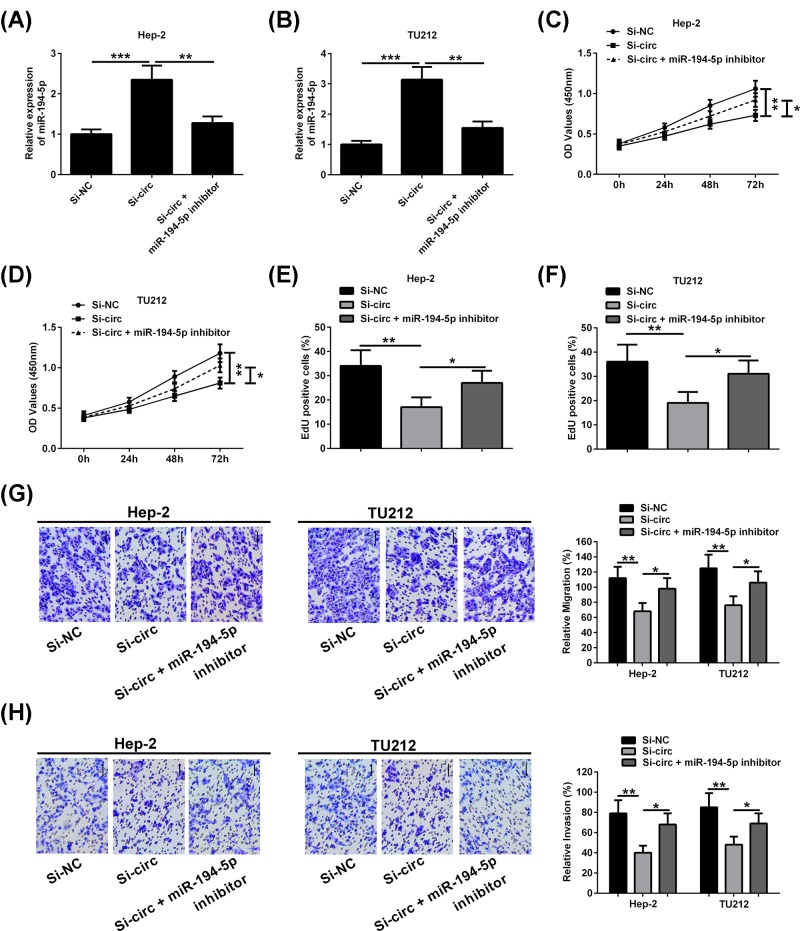
Down-regulation of miR-194-5p attenuates the inhibitory effect of hsa_circ_0023028 silencing on LCa cell proliferation, migration, and invasion (**A,B**) Hep-2 and TU212 cells were transfected with si-NC, si-circ, or si-circ + miR-194-5p inhibitor. miR-194-5p level was detected. (**C,D**) CCK-8 assay showed the cell viability of Hep-2 and TU212 cells transfected with si-NC, si-circ, or si-circ + miR-194-5p inhibitor. (**E,F**) EdU assay showed the proliferation ability of Hep-2 and TU212 cells transfected with si-NC, si-circ, or si-circ + miR-194-5p inhibitor. (**G,H**) The migration and invasion of Hep-2 and TU212 cells transfected with si-NC, si-circ, or si-circ + miR-194-5p inhibitor were determined using transwell migration and invasion assays, respectively. *P<0.05 and **P<0.01.

We further explored the potential involvement of miR-194-5p in mediating the effects of hsa_circ_0023028. Using an miR-194-5p inhibitor, we evaluated its ability to attenuate the effect of hsa_circ_0023028 silencing on the proliferation, migration, and invasion abilities of LCa cells. hsa_circ_0023028 silencing resulted in the upregulation of miR-194-5p (Figure 6A,B). CCK-8 assay showed that the viability of Hep-2 and TU212 cells was higher in the si-circ + miR-194-5p inhibitor group than in the si-circ group. Meanwhile, an increased number of EdU-positive cells was observed in the si-circ + inhibitor group as compared with the si-circ group, as indicated by the CCK-8 (Figure 6C,D) and EdU assays (Figure 6E,F). Moreover, transwell migration and invasion assays revealed that si-circ-mediated inhibition of cell migration and invasion in Hep-2 and TU212 cells was remarkably blocked by down-regulation of miR-194-5p (Figure 6G,H).

## Discussion

Currently, there are several studies describing the roles of circRNAs in LCa. Fan et al. [23] identified 506 differentially expressed circRNAs in LSCC. They found that hsa_circ_0044520 and hsa_circ_0044529 expression levels were up-regulated in LSCC [23]. Wu et al. [24] found that 3715 circRNAs were differentially expressed in CD133^+^CD144^+^ cancer stem cells derived from human LSCC cells. Among these circRNAs, hg19_circ_0005033 was up-regulated, and down-regulation of hg19_circ_0005033 repressed the proliferation, migration, and invasion of these CD133^+^CD144^+^ cancer stem cells [24]. With regard to the normal tissues, hsa_circ:chr20:31876585-31,897,648 was validated to be down-regulated in LSCC tissues, indicating that it might serve as a novel tumor suppressor in LSCC [25]. In summary, previous literature shows that circRNAs play an important role in tumorigenesis. However, the specific circRNAs in LCa are just beginning to be characterized. In the present study, we first identified the function of hsa_circ_0023028 in LCa. We found that hsa_circ_0023028 could promote the proliferation, migration, and invasion abilities of LCa cells, indicating that hsa_circ_0023028 functions as a potential oncogene in LCa.

miRNAs have been extensively discussed in human cancers and serve as crucial regulators in numerous physiological processes. miR-194-5p has been shown to play an important role in tumorigenesis, although its role may vary in different cancers. Su et al. [26] identified the involvement of miR-194-5p in glioma. They demonstrated that miR-194-5p was down-regulated in glioma, wherein it functioned as a tumor suppressor to repress the malignant biological behaviors of glioblastoma stem cells [26]. Moreover, long noncoding RNA X-inactive specific transcript-induced down-regulation of miR-194-5p was shown to promote the proliferation, migration, and invasion of hepatocellular carcinoma cells by suppressing mitogen-activated protein kinase 1 expression [20]. Through targeted inhibition of lysosome-associated membrane protein-2, miR-194-5p repressed the cell proliferation ability of sunitinib-resistant ACHN cells in the presence of sunitinib [27]. Additionally, miR-194-5p was shown to be up-regulated in breast cancer tissues, and silencing of miR-194-5p repressed the cell proliferation, migration, and invasion of MCF-7 cells by promoting SRY-box 17 expression through the Wnt/β-catenin signaling pathway [28]. A recent study also showed that miR-194-5p was underexpressed in LSCC tissues and low expression of miR-194-5p was correlated with T stages, clinical stages, recurrence, and poor prognosis in LSCC. Furthermore, enhanced expression of miR-194-5p has been shown to repress the malignant phenotypes of LSCC by targeting Wee1 [21]. Li et al. [21] have demonstrated that miR-194-5p is a tumor suppressor in LSCC and they identified Wee1 as its downstream target gene. However, to date, the upstream regulatory mechanism of abnormal expression of miR-194-5p in LCa remains unclear. In the present study, down-regulation of miR-194-5p was validated in LCa tissues and cells, which was consistent with the previous study by Li et al. [21]. A negative correlation between miR-194-5p and hsa_circ_0023028 expression was observed in LCa tissues, suggesting the involvement of miR-194-5p in mediating the effects of hsa_circ_0023028 in LCa.

The interaction between ncRNA molecules is a novel and important regulatory mechanism in tumorigenesis [29]. Recently, the potential of circRNAs as gene regulators has been emphasized. It is noteworthy that circRNAs function as competitive endogenous RNAs to regulate gene expression through sponging miRNAs [30]. Due to the presence of multiple miRNA binding sites, circRNAs can serve as competitive inhibitors that inhibit the ability of miRNAs to bind their target mRNAs [31]. An example of this is the circRNA circMTO1, which harbors the miR-9 binding sites. circMTO1 serves as an miR-9 sink to increase the expression of the miR-9 target p21, thereby inhibiting the proliferation and invasion of hepatocellular carcinoma cells [32]. Li et al. [21] have shown the role of miR-194/Wee1 axis in regulation of the proliferation, migration, and invasion in LSCC cells. Based on [21], we delved more deeply into the regulatory mechanism of miR-194-5p in the proliferation, migration, and invasion of LCa cells. We reported, for the first time, the identification of hsa_circ_0023028 as an miR-194-5p sink, and demonstrated that hsa_circ_0023028 promoted the proliferation and metastasis of LCa cells by sponging miR-194-5p.

In conclusion, we first reported the up-regulation of hsa_circ_0023028 in LCa. Moreover, we showed that hsa_circ_0023028 promotes the proliferation and metastasis of LCa cells by sponging miR-194-5p. Hence, our research provides a better understanding of the relationship between circRNA deregulation and carcinogenesis.

## Abbreviations

ACHN: renal adenocarcinoma cells
CCK-8: cell counting kit-8
circRNA: circular RNA
EdU: 5-Ethynyl-2′-deoxyuridine
FBS: fetal bovine serum
GAPDH: glyceraldehyde-3-phosphate dehydrogenase
LCa: laryngeal cancer
LSCC: laryngeal squamous cell carcinoma
miRNA: microRNA
miR-NC: miRNA negative control
ncRNA: noncoding RNA
qRT-PCR: quantitative real-time polymerase chain reaction
si-NC: siRNA negative control
siRNA: small interfering RNA
TNM: tumor-node-metastasis
UTR: untranslated region
